# Transcatheter management of cardiogenic shock in severe aortic stenosis

**DOI:** 10.3389/fcvm.2026.1803093

**Published:** 2026-04-29

**Authors:** Francesca Maria Di Muro, Gianmarco Sarto, Valeria Raona, Angelo Oliva, Mauro Chiarito, Angelo Silverio, Sebastiano Sciarretta, Tiziana Attisano, Carmine Vecchione, Gennaro Galasso

**Affiliations:** 1 Department of Medicine, Surgery, and Dentistry, University of Salerno, Salerno, Italy; 2 Cardiology Unit, Cardiovascular and Thoracic Department, University Hospital “San Giovanni di Dio e Ruggi d'Aragona”, Largo Città di Ippocrate, Salerno, Italy; 3 Department of Medical-Surgical Sciences and Biotechnologies, Sapienza University of Rome, Latina, Italy; 4 Department of Cardiology, ICOT Istituto Marco Pasquali, Latina, Italy; 5 Department of Biomedical Sciences, Humanitas University, Milan, Italy; 6 The Zena and Michael A. Wiener Cardiovascular Institute, Icahn School of Medicine at Mount Sinai, New York, NY, United States; 7 Department of Cardiology, IRCCS NeuroMed, Pozzilli, Italy

**Keywords:** balloon aortic valvuloplasty, cardiogenic shock, mechanical circulatory support, severe aortic stenosis, transcatheter aortic valve implantation

## Abstract

Management of cardiogenic shock (CS) in the setting of severe aortic stenosis (AS) remains clinically challenging, owing to the coexistence of fixed valvular obstruction, limited myocardial reserve, and rapidly evolving end-organ hypoperfusion. In this context, early identification of patients unlikely to recover with medical therapy alone and timely transition to definitive valve intervention are critical determinants of outcome. Despite growing clinical experience, decision-making in this setting remains heterogeneous and largely informed by observational evidence. Herein, in the present review, we synthesize contemporary evidence on transcatheter strategies for AS-related CS, with emphasis on clinical decision-making during the acute phase. A physiology-driven approach to early assessment and hemodynamic stabilization is outlined, integrating echocardiography, invasive monitoring, and tailored pharmacologic support to inform escalation pathways. Mechanical circulatory support (MCS) is discussed as a selective, time-limited strategy to achieve stabilization and facilitate procedural planning, while accounting for the unique physiological limitations imposed by fixed valvular obstruction. Available data on balloon aortic valvuloplasty and urgent or emergent transcatheter aortic valve implantation (TAVI) are examined with respect to procedural feasibility, early outcomes, and their positioning within bridging or definitive treatment pathways in CS. Collectively, this work proposes a pathway-oriented framework for AS-related CS that prioritizes early recognition, coordinated multidisciplinary decision-making, and timely progression to definitive valve intervention, tailored to patient risk profile and institutional expertise.

## 1 Introduction

Cardiogenic shock (CS) complicating severe aortic stenosis (AS) represents one of the most lethal and time-critical presentations in contemporary cardiovascular medicine. Fixed left ventricular outflow tract (LVOT) obstruction, limited myocardial reserve, and systemic hypoperfusion interact to create a pathophysiological state in which conventional shock therapies are frequently insufficient, and early mortality remains substantial (1, 2).

The advent of transcatheter therapies has deeply transformed the therapeutic landscape by enabling definitive relief of valvular obstruction in patients who are often unsuitable for surgical intervention in the acute setting (3–8). Nonetheless, management remains inherently complex, requiring prompt diagnostic confirmation, physiology-guided hemodynamic support, and early recognition of patients unlikely to recover without timely valve intervention. Within this evolving framework, the expanding role of mechanical circulatory support (MCS) has broadened the range of bridging and procedural strategies; however, its use in severe AS is constrained by distinct hemodynamic considerations and necessitates clearly defined escalation and exit pathways. Despite increasing clinical experience, evidence guiding the optimal timing and sequencing of transcatheter interventions in AS-related CS remains limited and predominantly observational, and the absence of randomized data—together with heterogeneity in shock phenotypes, anatomical complexity, and institutional expertise—continues to drive substantial variability in contemporary practice (9).

**Figure 1:**
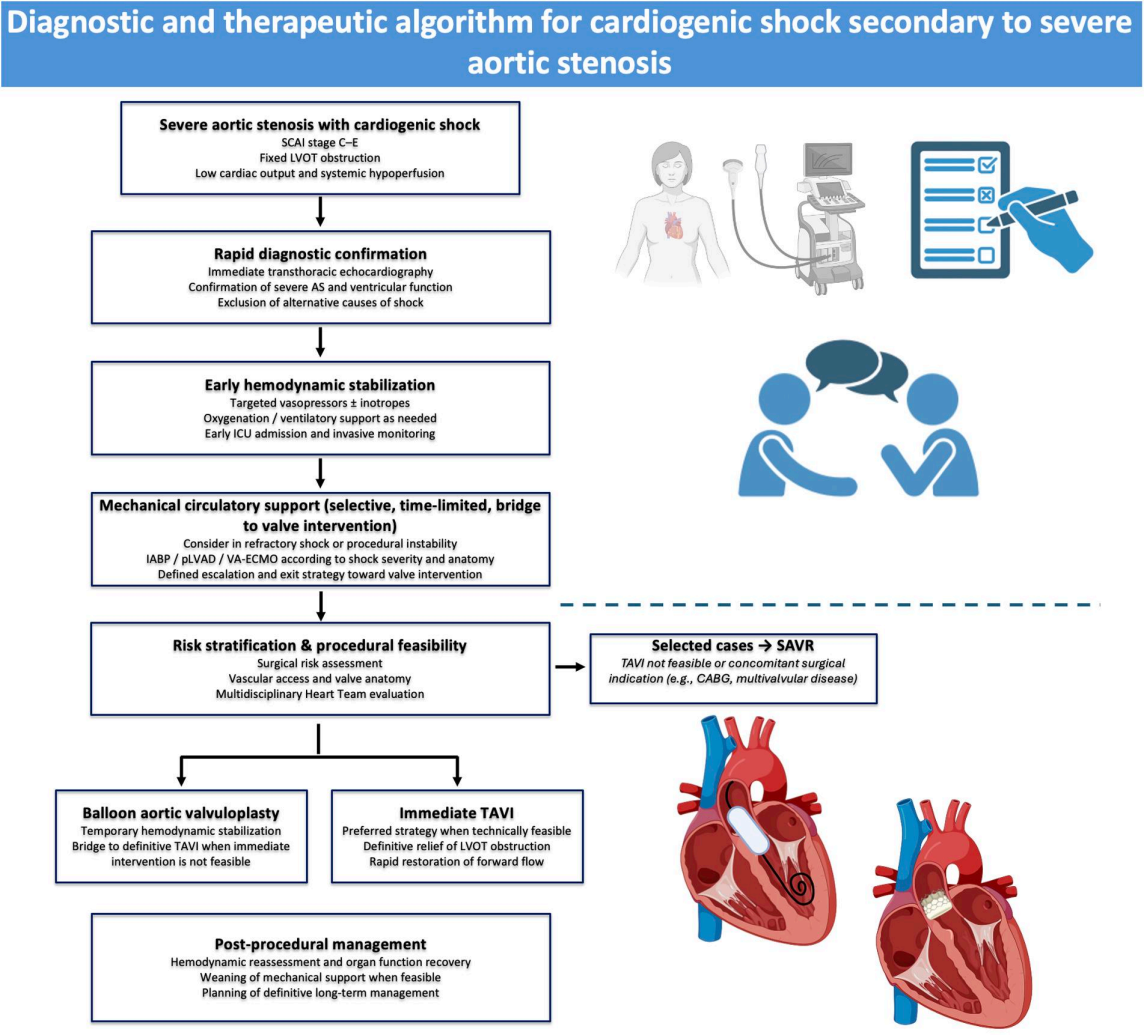
Central illustration. Diagnostic and therapeutic algorithm for cardiogenic shock in patients with severe aortic stenosis, outlining a stepwise approach from early diagnosis and stabilization to Heart Team–guided valve intervention and post-procedural management.

In this review, we synthesize available evidence and expert consensus on the transcatheter management of CS complicating severe AS. We outline principles of early clinical assessment and hemodynamic support, examine the role of MCS as a bridge to intervention, and review the available data supporting balloon aortic valvuloplasty (BAV) and urgent or emergent transcatheter aortic valve implantation (TAVI), proposing a pathway-oriented management strategy aimed at minimizing delays to definitive therapy while mitigating procedural risk in this high-risk population (Figure 1).

## 2 Clinical assessment and early stabilization

CS in the setting of severe AS represents an advanced stage of hemodynamic deterioration in which fixed LVOT obstruction critically limits compensatory responses to acute circulatory stress. Clinical presentation may result from progressive decompensation of chronic valvular disease or from abrupt hemodynamic collapse triggered by superimposed acute cardiovascular insults, including myocardial ischemia, tachyarrhythmias, or acute volume shifts (10).

Although CS complicates only a small proportion of hospitalizations for severe AS (approximately 1.6%–3.2%), its onset is typically associated with advanced shock severity. Most patients fulfill criteria corresponding to Society for Cardiovascular Angiography and Interventions (SCAI) shock stages C through E, reflecting sustained hypotension with objective evidence of hypoperfusion and a markedly increased short-term mortality risk (11–14). Early recognition of this transition is crucial, as delays in diagnosis or intervention are associated with rapid clinical deterioration and adverse outcomes (15, 16).

From a clinical standpoint, CS is defined by persistent hypotension (systolic blood pressure <90 mmHg for more than 30 min) despite adequate preload, accompanied by signs of systemic hypoperfusion (12). Physical examination should prioritize the assessment of global perfusion, respiratory status, and evidence of congestion or low-output failure. In advanced shock states, classic auscultatory findings of AS are frequently attenuated due to reduced transvalvular flow, limiting the diagnostic reliability of physical examination alone (17, 18).

Point-of-care cardiac ultrasound plays a central role in the initial evaluation, allowing rapid exclusion of alternative causes of hemodynamic collapse, such as acute myocardial ischemia with severe ventricular dysfunction, cardiac tamponade, massive pulmonary embolism, or dynamic LVOT obstruction (19). Findings suggestive of critical valvular obstruction should prompt urgent comprehensive transthoracic echocardiography to confirm AS severity and establish the presence of a valvular emergency (20). Echocardiographic assessment integrates valve area, Doppler gradients, stroke volume, ventricular function, and leaflet morphology. Interpretation requires careful contextualization, as low-flow states may underestimate gradients, while inotropic support can artifactually increase them (20, 21).

Electrocardiography plays an adjunctive role by identifying ischemia or tachyarrhythmias, particularly atrial fibrillation with rapid ventricular response, which is poorly tolerated in AS (22). The hypertrophied LV is particularly vulnerable to ischemia, arrhythmias, and hypovolemia, and abrupt loss of atrial contraction may precipitate hemodynamic deterioration. In unstable patients, urgent rhythm control is recommended (23).

Finally, laboratory assessment provides complementary prognostic and monitoring information. Markers of tissue hypoperfusion and end-organ dysfunction, including serum lactate, mixed venous oxygen saturation (SvO_2_), veno-arterial carbon dioxide difference (ΔCO_2_), renal and hepatic markers, and troponin and natriuretic peptides, should be serially assessed to guide escalation and evaluate response to therapy. Elevated lactate levels are strongly associated with mortality, while early lactate clearance has been linked to improved outcomes (24).

Hemodynamic stabilization should begin promptly, with continuous clinical evaluation, supported by serial hemodynamic assessment. In patients with severe AS, who exhibit marked preload dependence and limited ability to increase stroke volume, fluid resuscitation should not be used as a primary therapy but may consist of cautious, small-volume fluid challenges to exclude concomitant hypovolemia, with close monitoring for signs of volume intolerance (25). Diuretic therapy should generally be avoided unless required for life-threatening pulmonary congestion. Vasodilators are usually contraindicated in the acute shock setting, as fixed LVOT obstruction prevents compensatory increases in CO and may precipitate severe hypotension (2).

In the absence of randomized trial data, when vasopressor support is required, norepinephrine should be used as the first-line agent, preferred over dopamine given its more favorable safety profile and lower arrhythmogenic potential (26). Although norepinephrine may increase systemic vascular resistance and transvalvular gradients, careful titration to the minimal effective dose to maintain mean arterial pressure is required (20). If tissue hypoperfusion persists despite restoration of perfusion pressure, dobutamine or adrenaline may be considered, particularly in patients not receiving chronic beta-blocker therapy, with continuous monitoring for tachyarrhythmias and ischemia (26). Excessive tachycardia should be avoided, as it increases myocardial oxygen demand and shortens diastolic filling time, further compromising ventricular filling and forward flow.

Airway and ventilatory management in this population must be approached with extreme caution. Positive-pressure ventilation can substantially impair venous return and reduce cardiac afterload through reflex vasodilation, while anesthetic induction during intubation carries a high risk of abrupt hemodynamic deterioration (27). When endotracheal intubation is unavoidable, careful pre-emptive hemodynamic optimization—using the lowest effective induction doses and anticipatory vasopressor support—is critical to mitigate the risk of cardiovascular collapse.

## 3 Mechanical circulatory support in aortic stenosis-related shock

In patients who remain hemodynamically unstable despite initial medical stabilization, MCS may be required as an adjunctive bridging strategy in AS–related CS, with the primary aim of rapidly restoring sufficient systemic perfusion to enable timely definitive valve intervention before the onset of irreversible multiorgan dysfunction. However, its use in this context presents distinct physiological and technical challenges, as fixed LVOT obstruction fundamentally alters both device feasibility and expected hemodynamic efficacy. Evidence to guide MCS selection and timing remains limited and is derived almost exclusively from observational studies and registry data, resulting in substantial heterogeneity in clinical practice and continued reliance on expert consensus (20).

Intra-aortic balloon pump (IABP) use in severe AS remains controversial. IABP primarily provides an increase in coronary perfusion during diastole and a reduction of LV afterload. In contrast to active forward-flow devices, however, these effects are intrinsically limited in the setting of severe, fixed valvular obstruction and do not reliably translate into improved forward flow. While large, randomized trials in unselected CS populations have demonstrated no survival benefit with routine IABP use, leading to downgraded guideline recommendations, particularly in MI-related shock, these findings may not be fully generalizable to AS-related shock (28, 29). Consequently, IABP employment can be considered as a temporary stabilizing strategy in severe AS, particularly in mixed shock phenotypes with concomitant ischemia or arrhythmias, or as a short bridge to valve intervention such as BAV or TAVI, rather than as definitive therapy (30).

Percutaneous left ventricular assist devices (pLVADs), particularly Impella (Abiomed Inc., Danvers, MA, USA), are often technically challenging in severe AS because device deployment requires crossing a heavily calcified and critically narrowed valve, with attendant risks of acute hemodynamic deterioration, hemolysis, calcium or thrombotic embolization, and valve injury. Accordingly, professional societies, including the Society for Cardiovascular Angiography and Interventions (SCAI), the American College of Cardiology (ACC), and the Society of Thoracic Surgeons (STS), list severe AS as a relative contraindication to Impella use. Current practice, therefore, favors either planned BAV before Impella placement or avoidance of pLVADs altogether when anatomy predicts poor device deliverability or flow limitation across the stenotic valve¹⁵. When successfully deployed—typically in experienced centers using combined BAV–Impella strategies—pLVADs can provide meaningful short-term hemodynamic support and facilitate timely definitive intervention (20, 31, 32). Of note, the hemodynamic support provided by the Impella microaxial flow pump might be pivotal in recovering from the stress caused by rapid pacing during BAV or TAVI.

Veno-arterial extracorporeal membrane oxygenation (VA-ECMO) offers immediate, full cardiopulmonary support and is often reserved for refractory shock or cardiac arrest. However, VA-ECMO increases LV afterload, which in severe AS may exacerbate pulmonary congestion and limit aortic valve opening (31). This trade-off underscores an ongoing area of debate, with consensus statements emphasizing the importance of concomitant LV unloading strategies, such as adjunctive pLVAD use when feasible, atrial septostomy, or left-atrial VA-ECMO configurations, to mitigate LV distension (20). In specialized centers, ECMO is increasingly used as a bridge to urgent BAV or TAVI when rapid deterioration precludes alternative stabilization strategies (7, 32).

In selecting an MCS strategy, experience from high-risk PCI underscores that patient selection is paramount, a principle that is equally applicable to TAVI in CS (33). Device choice should be individualized based on integrated echocardiographic and invasive hemodynamic assessment, incorporating ventricular function, valve anatomy, valve and arterial calcification burden, and the presence of dynamic gradients, reflecting the broader paradigm of multimodal cardiovascular assessment to guide individualized decision-making (34). Accordingly, current expert recommendations caution against indiscriminate MCS deployment and emphasize the importance of institutional expertise, multidisciplinary coordination, and the definition of clear escalation and exit strategies (2, 26).

Beyond rescue therapy, MCS may also be required intraprocedurally during TAVI to manage acute hemodynamic deterioration. A systematic review of observational data evaluating periprocedural cardiopulmonary bypass or VA-ECMO during TAVI reported that mechanical support was required in approximately 4% of procedures, most commonly as an emergent response to rapid hemodynamic collapse, severe AR, ventricular arrhythmias, or coronary obstruction. In these cohorts, short-term mortality approached 30%, and 1-year mortality exceeded 50%, reflecting the critical clinical profile of these patients. However, the use of CPB or VA-ECMO is not without risk and may introduce additional complications—particularly vascular and bleeding events related to large-bore access—superimposed on those already associated with the TAVI procedure itself (20, 35).

Importantly, MCS should be regarded as a supportive and time-limited strategy, rather than a therapeutic endpoint. Its value lies in stabilizing selected patients and facilitating informed procedural decision-making, while prolonged support in the absence of a definitive treatment pathway risks exposing patients to device-related complications without meaningful clinical benefit. Ultimately, outcomes in AS–related CS depend on timely identification of patients likely to benefit from temporary mechanical support versus those requiring immediate transition to definitive valve intervention, as delayed resolution of the underlying obstruction is associated with progressive organ dysfunction and futility.

## 4 Balloon aortic valvuloplasty and urgent/emergent TAVI in cardiogenic shock and severe aortic stenosis

Against this background, definitive management of CS in severe AS hinges on prompt relief of the valvular obstruction, which is the only intervention capable of preventing the progressive decline in organ perfusion and function driven by persistently reduced CO. According to current European guidelines, surgical aortic valve replacement (SAVR) is generally recommended in patients at low surgical risk (<4%, assessed by STS-PROM or EuroSCORE II) (36). However, in patients presenting with CS, surgical risk scores almost invariably exceed this threshold, precluding classification as low-risk by any validated scoring system and effectively shifting treatment strategies toward percutaneous approaches (37, 38).

In patients with severe AS who cannot be deemed at low surgical risk, current guidelines and consensus documents identify two principal transcatheter options: TAVI or BAV, with BAV typically used as a temporizing strategy and as a bridge to definitive valve replacement (either TAVI or SAVR) (20, 38). However, there is no clear evidence to support the preferential use of one strategy over the other in the setting of CS, and no randomized trials have compared emergency TAVI with an initial BAV-first approach in this population. Accordingly, selection among available strategies should be guided by a comprehensive Heart Team evaluation, integrating clinical status, anatomical suitability, procedural feasibility, and longer-term management considerations, including life expectancy (36).

Despite the absence of randomized data, both American and European guidelines reflect a progressive shift toward primary TAVI in anatomically suitable patients. Specifically, the ACC/AHA guidelines acknowledge that while BAV may provide temporary stabilization, its contemporary use has declined owing to the high procedural success of immediate TAVI even in very high-risk patients. Similarly, the ESC/EACTS 2025 guidelines report that BAV has been largely replaced by TAVI in recent years, given its limited durability and enhanced risk of acute severe aortic regurgitation after balloon dilatation (36, 39). Nonetheless, a role for SAVR may persist in selected patients who achieve hemodynamic stabilization—often after an initial BAV strategy—and in whom anatomical features or concomitant cardiac conditions favor surgical correction, such as multivalvular disease or the need for concomitant CABG. Key observational studies and contemporary meta-analyses informing urgent/emergent BAV and TAVI in AS-related CS are summarized in Table 1.

**Table 1:** Evidence on urgent/emergent TAVI in severe AS with cardiogenic shock.

Study	Design/setting	N	Population	Intervention	In-hospital/30-d outcomes	90-d/1-y (or longer)	Key complications	Conclusions
NHLBI Balloon Valvuloplasty Registry (1991)	Prospective registry (USA)	674	Adults with severe AS undergoing percutaneous BAV	BAV	30-d cumulative mortality 14% (92/643)	The study did not report outcomes beyond mortality.	Complications before discharge included transfusion (23%), vascular surgery (7%), cerebrovascular accident (3%), other systemic embolus (2%), MI (2%), acute tubular necrosis (1%), or cardiac surgery (1%).	BAV provides symptomatic and hemodynamic improvement but carries substantial early morbidity and 30-day mortality in high-risk patients; it is primarily a palliative or bridge therapy.
Cribier et al. (40)	Case report/letter	10	Severe AS and CS	BAV	Early mortality: 20%	Six patients had subsequent AVR a mean of 5 months after valvuloplasty. Two patients who refused surgery were alive without HF symptoms at 48 and 24 months, respectively.	Transient electromechanical dissociation during first balloon inflation requiring brief cardiac massage (*n* = 1). Restenosis with recurrent shock (death during repeat catheterization, *n* = 1). Death at 3 weeks from gastrointestinal bleeding (*n* = 1).	BAV is associated with transient hemodynamic stabilization but high early mortality; its role is primarily as a bridge to definitive therapy or decision-making.
Moreno et al. (59)	Single-center case series	21	Critical AS with CS	BAV	In-hospital mortality 43% (9/21)	27-month survival 35 ± 11%	Vascular complications (*n* = 5); stroke (*n* = 1); cholesterol emboli (*n* = 1); severe AR requiring AVR (*n* = 1)	Emergency BAV can be performed in CS due to critical AS but is associated with high in-hospital mortality and complications.
D’Ancona et al. (60)	Prospective/Single center	21	CS patients	Transapical TAVR	30-d mortality 33.3%; predictors: CO <3.0, low CPI, renal dysfunction, ventilation	1-y survival 59.3% (emergent) vs. 82.7% (elective)	Severe AKI	Transapical TAVR is feasible in CS, but early mortality remains high in the most unstable patients.
Saia et al. (43)	Single-center cohort (2000−2010)	415 (CS subgroup *n* = 23)	BAV for bridge/palliation; CS subgroup	BAV	In-hospital mortality 56.5% in CS subgroup (overall 5.1%)	Shock subgroup: 1-y mortality 70.7%; 2-y mortality 80.4%	Overall: stroke 0.5%; major vascular 2.2%; life-threatening bleeding 1.5%	BAV is relatively safe overall, but outcomes are poor when performed in CS.
Theiss et al. (44)	Post hoc analysis/ICU case series (2010−2012)	18	Severe AS with CS (*n* = 10) or acute decompensation (*n* = 3); other indications (*n* = 5)	BAV (some later transfemoral TAVI)	In-hospital mortality: 27.8% (5/18) (deaths within 24 h: 4 same-day, 1 at 24 h)	Follow-up: Not reported (authors report 8 patients weaned from catecholamines and alive at time of writing) Definitive therapy: transfemoral TAVI in 4 patients	Post-BAV AR: none 2; mild 13; moderate 2; moderate-to-severe 1 Procedural success: 100% (reported)	BAV can be used as bail-out therapy in CS or acute decompensation when definitive AVR or TAVR is not immediately feasible.
Frerker et al. (49)	Retrospective/Single center	27	CS with decompensated AS	TAVR (urgent vs. elective)	Device success 88.9% (24/27); 3 deaths within 72 h of valve deployment and 6 additional deaths within 30 days → 30-day mortality 33.3% (vs. 7.7% elective).	Estimated 1-year survival 59.3% (emergent) vs. 82.7% (elective); 30-day landmark analysis showed similar 1-y survival once patients survived 30 days.	Cardiopulmonary bypass 25.9%; stroke 3.7%; major bleeding 3.7% (life-threatening 0%); major vascular 3.7% (minor vascular 29.6%); new pacemaker 11.1%; AKI stage 3 29.6% (4 patients temporarily dialysis-dependent).	Emergency TAVR is a reasonable rescue therapy in CS due to decompensated AS, with excess mortality largely confined to the first 30 days.
Landes et al. (61)	Retrospective/Single center	27	ADHF from severe AS vs. elective	Urgent TAVR vs. emergent BAV strategy	Procedural mortality 8.7% (TAVR) vs. 20.3% (BAV)	Elective TAVR after BAV: 30-d CV mortality 15.6%	Vascular complications, stroke	Urgent TAVR is feasible in acute decompensated severe AS and may be used as definitive therapy during the index admission; outcomes remain worse than elective cases due to baseline risk.
Bongiovanni et al. (53)	Retrospective/Multicenter	141 (emergency TAVR 23; emergency BAV 118; elective TAVR after emergency BAV 32)	Decompensated AS requiring emergency intervention: emergency TAVR vs. emergency BAV -> elective TAVR	Emergency TAVR vs. emergency BAV (bridge-to-TAVR)	30-d CV mortality 23.8% (TAVR) vs. 33.0% (BAV)	Secondary TAVR after BAV still high risk	Vascular complications, stroke	Emergency TAVR and emergency BAV strategies are both associated with high early mortality in decompensated AS; definitive TAVR after emergency BAV remains high risk.
Debry et al. (46)	Retrospective/Multicenter (2 centers, France)	44	CS related to severe AS Hypotensive CS (*n* = 31) vs. non-hypotensive CS (*n* = 13)	Urgent BAV (bridge-to-AVR/TAVR in selected)	30-day (1-month) mortality: 47%	1-year composite all-cause death or recurrent CS: 75% 1-year mortality: 70% (*n* = 31) Staged TAVR/SAVR: 27% (12/44), median 79 days post-BAV	Univariate predictors of 1-y death/recurrent CS: dobutamine >5 mcg/kg/min; BAV >48 h after inotrope start (time-to-BAV concept)	Urgent BAV can be performed as rescue therapy in CS due to severe AS, but 1-year mortality remains high; earlier BAV (within 48 h of inotrope initiation) is associated with better outcomes.
Eugène et al. (41)	Single-center cohort (France; 2008−2016)	40	Severe AS with CS (*n* = 17) or refractory pulmonary edema (*n* = 23)	Rescue PBAV ± staged SAVR/TAVI	Procedural death: 0 Early death: 30% (12/40)	Bridged to SAVR/TAVI among survivors: 57% (16/28) 2-year estimated survival: SAVR 71 ± 17%; TAVI 36 ± 19%; PBAV alone 8 ± 8%	The study did not report outcomes beyond mortality.	Rescue PBAV can stabilize patients with CS or refractory pulmonary edema and improve survival when followed by definitive SAVR or TAVR.
Kolte et al. (62)	Retrospective/Multicenter	3,952	Urgent/emergent vs. elective TAVR in CS	TAVR	Device success slightly lower (92.6 vs. 93.7%); 30-d mortality higher (8.7 vs. 4.3%)	1-y mortality higher (29.1 vs. 17.5%)	AKI/dialysis	Urgent or emergent TAVR is feasible but is associated with higher AKI and higher 30-day and 1-year mortality than elective TAVR.
Huang et al. (63)	Retrospective/Single center	26	Emergency TAVR in decompensated valve disease and CS	TAVR	In-hospital mortality 19.4%; predictors: pulmonary artery pulsatility index ≤ 1.8, CPR, MCS post-TAVR	1-y survival 61%; 2-y survival 55.9%	AKI and dialysis	Emergency TAVR is feasible in extreme-risk decompensated patients; preserved right-heart function and early mechanical circulatory support are associated with better survival.
Varela et al. (45)	Retrospective/Single center (Portugal; CICU)	14	CS with severe AS Emergent (*n* = 9) vs. urgent (*n* = 5)	BAV (emergent vs. urgent)	In-hospital mortality: Not reported 30-day mortality: 21.4% (3/14)—Emergent: 33% (3/9)—Urgent: 0% (0/5)	Definitive AVR/TAVI after BAV: 6/14—Emergent: TAVI 4; SAVR 1—Urgent: TAVI 1 Long-term survival: reported per patient (incomplete)	Clinically significant AR: 0 Major post-procedure complications: 0 (reported)	BAV can be used as a rescue strategy in CS due to severe AS; short-term mortality is driven by baseline severity, and survivors may be bridged to definitive AVR.
Bandyopadhya et al. (64)	Retrospective/Multicenter	2,136	Urgent BAV vs. urgent TAVR	BAV vs. TAVR	No in-hospital mortality difference	The study did not report outcomes beyond mortality.	TAVR: pacemaker, bleeding, vascular	In a national inpatient analysis, urgent TAVR and urgent BAV had similar in-hospital mortality after propensity matching, with differing complication profiles.
Fraccaro et al. (57)	Retrospective/Multicenter	51	Severe AS and CS	TAVR	Device success 94.1%; 30-d mortality 11.8%	1-y mortality 25.7%	AKI 34%; stroke 2%; vascular 5.9%	TAVR is feasible in CS shock due to severe AS or degenerated bioprosthesis, with high procedural success and acceptable 1-year outcomes.
Masha et al. (14)	Retrospective/Multicenter	2,220	CS vs. non-CS undergoing TAVR	TAVR	30-d mortality 19.1% vs. 4.9%; success >90%	The study did not report outcomes beyond mortality.	Higher complications overall in CS	TAVR is feasible in CS, but early mortality increases with greater shock severity despite high procedural success.
Piriou et al. (58)	Retrospective/Multicenter	38	Rescue TAVR in CS and AS	TAVR	30-d mortality 7.9%	1-y mortality 21.1%; 29% rehospitalization	AKI, pacemaker; LBBB linked to mortality	Rescue TAVR is feasible in CS with low procedural mortality and acceptable 1-year survival; acute kidney failure and conduction disturbances are common.
Steffen et al. (50)	Retrospective/Single center	47	Emergent (CS/non-CS) vs. elective	TAVR	Elective mortality 5.3%; critically ill higher device failure	90-d mortality: CS 42.6% vs. non-CS 15.9%	Mechanical ventilation, hemofiltration, systemic inflammation, and hypotension were associated with increased mortality.	Emergency TAVI in critically ill patients has higher 90-day mortality, especially with CS, but survivors beyond 90 days have outcomes similar to elective TAVI.
Castelo et al. (65)	Retrospective/single center (Portugal; 2018–2021)	298 (urgent *n* = 79; elective *n* = 219)	Urgent vs. elective	TAVR	In-hospital mortality: 25.3% vs. 15.1%; 30-d CV mortality: 17.5% vs. 4.0%	Follow-up: 12 ± 5 mo; 1-y mortality and HF hospitalization: 21.1% vs. 13.5%; 1-y NYHA III-IV: 16.4% vs. 6.0%	Life-threatening bleeding: 11.5% vs. 4.1%; vascular: 11.5% vs. 4.6%	Urgent TAVI is associated with worse unadjusted short-term outcomes than elective TAVI; after risk adjustment, differences largely reflect baseline clinical risk.
Goel et al. (3)	Retrospective/Multicenter	4,952	Severe AS and CS treated with TAVR	TAVR	Procedural success: 97.9%; in-hospital mortality and 30-day mortality were higher in patients with CS than in those without.	1-y mortality similar after surviving 30 ds; QoL improved	Dialysis/MCS/AKI worsen mortality	TAVR in CS has high early mortality, but patients who survive beyond 30 days have similar 1-year survival and substantial functional improvement.
Llah et al. (51)	National Inpatient Sample (USA, 2016–2020); propensity matched	6,970 matched (3,485 TAVR vs. 3,485 BAV)	Severe AS with CS: direct TAVR vs. urgent BAV	Direct TAVR vs. BAV	In-hospital death: 17.8% (TAVR) vs. 38.9% (BAV) MI: 12.3% vs. 32.4% Acute CVA: 6.17% vs. 3.44% Primary composite (death/CVA/MI): 36.8% vs. 56.8%	Long-term outcomes: Not reported (NIS provides in-hospital only)	Heart block needing PPM: 11.9% (TAVR) vs. 6.03% (BAV) Primary safety composite (transfusion/vascular/PPM): 27.4% vs. 21.4% Bleeding (transfusion) and vascular complications: no significant difference (exact rates Not reported)	Direct TAVR in CS is associated with lower in-hospital mortality and myocardial infarction than BAV, but higher rates of stroke and new pacemaker implantation.
Ismayl et al. (66)	Nationwide observational (NRD USA; 2016–2021)	16,161 (TAVR *n* = 6,470; SAVR *n* = 9,691)	AS and CS: TAVR vs. SAVR	TAVR vs. SAVR	In-hospital mortality: 15.1% vs. 15.4%; stroke: 5.6% vs. 8.2%; AKI: 53.1% vs. 58.5%; major bleeding: 2.9% vs. 5.1%; vascular: 9.8% vs. 6.7%	90-d all-cause readmission: 14.3% vs. 11.2% (aHR 1.21, 95% CI: 0.98–1.47); HF readmission: similar	MI: 9.5% vs. 8.9%; PPM: 7.2% vs. 7.5%	In a national database analysis, TAVR and SAVR had similar in-hospital mortality and 90-day readmissions; TAVR had lower stroke, AKI, and bleeding but higher vascular complications.
Nair et al. (7)	Retrospective/single center (CICU; 2010–2021)	199	AS and CS treated during index hospitalization: medical (*n* = 81) vs. BAV (*n* = 46) vs. TAVR (*n* = 24) vs. SAVR (*n* = 48)	Medical vs. BAV vs. TAVR vs. SAVR	In-hospital mortality: highest in medical vs. any intervention; BAV vs. TAVR/SAVR: non-significant difference; 30-d mortality: 50% vs. 26% vs. 4% vs. 16%	90-d mortality after BAV: ≈50% if not followed by AVR ≤90 d; 1-y mortality: 63% vs. 64% vs. 19% vs. 20%	The study did not report outcomes beyond mortality.	Definitive TAVR or SAVR is associated with the best short- and long-term outcomes in severe AS with CS; BAV benefit is time-limited unless followed by definitive AVR within 90 days.
Ferrera et al. (67)	Retrospective observational study of BAV in a contemporary cohort from Spain (2016–2022).	431	Severe AS and CS (ICU admissions) treated with: Medical (*n* = 260) vs. TAVR (*n* = 107) vs. SAVR (*n* = 64) (BAV-only cases excluded)	TAVR vs. SAVR vs. medical	In-hospital mortality: medical 60.8%; TAVR 20.6%; SAVR 32.8% (overall 46.6%) 30-day readmission: medical 7.9%; TAVR 10.8%; SAVR 0%	The study did not report outcomes beyond mortality.	Complications (during hospitalization): Acute renal failure 21.5% (TAVR) vs. 14.1% (SAVR) vs. 11.2% (medical) Blood transfusion 24.3% vs. 26.6% vs. 12.3% Peripheral vascular disease 10.3% vs. 10.9% vs. 3.5% Stroke 5.6% vs. 0% vs. 1.2% AV block 8.4% vs. 0% vs. 0% Any circulatory support device 11.2% vs. 25% vs. 11.5%	TAVR is associated with lower in-hospital mortality than SAVR or medical therapy in severe AS with CS; AKI, pacemaker implantation, and mechanical circulatory support predict worse prognosis.
Jabri et al. (68)	Systematic review and meta-analysis	5 studies; 26,283	TAVR in CS vs. no-CS	TAVR	30-d mortality: OR 3.41 (95% CI: 2.01–5.76); 30-d vascular: OR 1.72 (1.54–1.92)	1-y mortality: OR 2.68 (0.53–13.46), NS	30-d stroke: OR 1.54 (0.57–4.15), NS; PPM: OR 0.75 (0.40–1.41), NS; in-hospital major bleeding: OR 2.45 (0.43–13.83), NS	TAVR performed in patients with CS is associated with higher 30-day mortality and major vascular complications than TAVR without shock, while 1-year mortality may be similar among survivors.
Dimitriadis et al. (52)	Systematic review and meta-analysis	6 studies; 21,020	AS and CS or ADHF: urgent TAVR vs. BAV	TAVR vs. BAV	In-hospital mortality: RR 0.53 (95% CI: 0.32–0.87); 30-d mortality: RR 0.51 (0.31–0.84)	Pooled mortality: in-hospital 10.4% vs. 21.7%; 30-d 11.8% vs. 27.6%	Across cohorts: MI 12.3% vs. 32.4%; stroke 6.2% vs. 3.4%; PPM 11.9% vs. 6.0% (selected studies)	In this meta-analysis directly comparing urgent TAVI versus BAV in CS or ADHF, urgent TAVI was associated with lower in-hospital and 30-day mortality.
Kühne et al. (56)	Systematic review and meta-analysis	17 studies; 2,811	Emergency TAVR vs. emergency BAV in CS due to severe AS	TAVR vs. BAV	In-hospital mortality: pooled estimate 11% (95% CI: 0.06–0.18) vs. 40% (95% CI: 0.28–0.54); 30-day mortality: pooled estimate 19% (95% CI: 0.17–0.20) vs. 39% (95% CI: 0.32–0.46)	1-year mortality: pooled estimate 29% (95% CI: 0.20–0.40) vs. 67% (95% CI: 0.58–0.74)	Bleeding: 12% (95% CI: 0.06–0.20) vs. 15% (95% CI: 0.10–0.21); major vascular complications: 8% (95% CI: 7–10) vs. 3% (95% CI: 0–0.23)	In this meta-analysis comparing emergency TAVR and emergency BAV in CS due to severe AS, emergency TAVR was associated with lower pooled in-hospital, 30-day, and 1-year mortality.

ADHF, acute decompensated heart failure; AKI, acute kidney injury; AR, aortic regurgitation; AS, aortic stenosis; AVR, aortic valve replacement; BAV, balloon aortic valvuloplasty; CI, confidence interval; CO, cardiac output; CPI, cardiac power index; CPR, cardiopulmonary resuscitation; CS, cardiogenic shock; CV, cardiovascular; CVA, cerebrovascular accident; HF, heart failure; HR, hazard ratio; ICU, intensive care unit; LBBB, left bundle branch block; LVEF, left ventricular ejection fraction; MCS, mechanical circulatory support; MI, myocardial infarction; NRD, Nationwide Readmissions Database; OR, odds ratio; PPM, permanent pacemaker; QoL, quality of life; RR, risk ratio; SAVR, surgical aortic valve replacement; TAVR, transcatheter aortic valve replacement.

### 4.1 Balloon aortic valvuloplasty in cardiogenic shock: evidence from clinical studies

BAV was among the earliest transcatheter options proposed for severe AS complicated by CS. Early experience, including the seminal reports by Cribier et al., demonstrated that BAV can acutely reduce transvalvular gradients and transiently increase CO, resulting in immediate hemodynamic improvement in critically ill patients (40). Subsequent case series confirmed these short-term effects, supporting the role of BAV as a rapid unloading intervention of the obstructed valve (41). However, the hemodynamic response to BAV appears highly dependent on baseline shock severity and myocardial reserve. In advanced shock states, valve opening alone may be insufficient to restore effective forward flow. Consistent with this concept, Buchwald et al. reported no significant improvement in systolic blood pressure or cardiac index following BAV, suggesting limited capacity of the failing myocardium to translate relief of valvular obstruction into meaningful circulatory recover (42).

Across contemporary cohorts, mortality following BAV in the setting of CS remains substantial. In-hospital mortality ranges from approximately 30% in earlier reports to over 70% in the most severe series, while 30-day and one-year mortality reach up to 47% and 70%, respectively (43–46). Variability in outcomes largely reflects differences in baseline clinical severity, timing of intervention, and the extent of associated organ dysfunction. Importantly, shock phenotype further modulates prognosis: Debry et al. demonstrated significantly worse survival and lower rates of successful bridging to definitive therapy among patients with hypotensive CS compared with those presenting without sustained hypotension.

Procedural complications are inconsistently reported across studies. Vascular complications occur in up to 20% of patients in selected series, while the incidence of severe acute aortic regurgitation is generally ≤5% (43–46). Although modest in absolute terms, this rate exceeds that observed in non-shock populations and likely reflects the hemodynamic instability and procedural urgency inherent to CS (47, 48).

Taken together, available evidence indicates that BAV provides rapid but typically transient hemodynamic benefit, while short- and long-term mortality remain high. In contemporary practice, BAV is therefore best considered a temporary stabilizing strategy, particularly when immediate definitive valve intervention is not feasible or when used early as a bridge toward valve replacement.

### 4.2 Emergency TAVI in cardiogenic shock: evidence from clinical studies

Emergency TAVI is a feasible and increasingly adopted definitive strategy in patients with severe AS presenting with CS. Early experience, including the initial reports by Frerker et al., demonstrated that emergency TAVI can be performed with high procedural success despite profound hemodynamic instability (49). Larger contemporary analyses have meaningfully expanded the evidence base. In a nationwide cohort of more than 2,200 patients, Masha et al. reported 30-day and 1-year mortality rates of 19% and 35%, respectively, with preprocedural cardiac arrest and the need for MCS emerging as strong predictors of adverse outcomes (14). Goel et al., analyzing the largest dedicated cohort to date (*n* = 5,006), observed lower mortality rates—12.9% at 30 days and 29.7% at 1 year—with baseline or in-hospital dialysis identified as the strongest independent predictor of death (3).

Additional insights derive from analyses of urgent or emergent TAVI cohorts not restricted to CS, which demonstrate a stepwise increase in mortality from elective to urgent and emergent presentations. These data support the concept that baseline hemodynamic severity at presentation, rather than procedural factors, is the main determinant of outcome. Accordingly, results from broader urgent or emergent cohorts should not be directly extrapolated to true CS, given the substantially worse outcomes observed in patients with overt shock compared with those presenting with acute decompensation without objective evidence of systemic hypoperfusion (50).

Direct comparative data between immediate TAVI and an initial BAV in patients with severe AS complicated by overt CS remain limited (7, 51). Nevertheless, available studies consistently demonstrate lower in-hospital and short-term mortality with immediate TAVI compared with BAV. These findings are further supported by pooled analyses of urgent-setting studies, which similarly show significantly lower in-hospital and 30-day mortality with urgent TAVI relative to BAV (7, 51–56). However, these comparisons should be interpreted cautiously given the inherent limitations and potential selection bias of observational datasets.

Peri-procedural complications are dominated by acute kidney injury, occurring in approximately 30% of patients, with 3%–4% requiring renal replacement therapy (3, 49, 57, 58). Major vascular events and clinically relevant bleeding remain relatively uncommon (≈3%–4%), while severe paravalvular regurgitation occurs in fewer than 5% of cases. Permanent pacemaker implantation rates approximate 10%, comparable to those observed in elective TAVI populations (3, 14, 50, 57, 58).

Taken together, current evidence supports primary TAVI as the preferred strategy when anatomical suitability, operator expertise, and institutional resources permit timely intervention. Nonetheless, BAV retains a complementary role when immediate TAVI is not feasible, either as a rapid stabilizing measure or as a bridge in centers without emergent TAVI capability.

## 5 Conclusions

CS complicating severe AS remains one of the most complex and time-critical scenarios in contemporary interventional practice. In this setting, clinical outcomes are increasingly driven by the timeliness of definitive restoration of valvular function rather than by supportive therapies alone. The evolution of transcatheter interventions has profoundly changed the therapeutic landscape, extending definitive treatment to patients previously considered unsuitable for acute intervention and reorienting management toward early, pathway-based valve correction. Mechanical circulatory support continues to play an important but adjunctive role within this continuum of care, primarily facilitating stabilization and transition to definitive therapy. Looking ahead, meaningful progress will require a more coherent alignment of clinical assessment, hemodynamic management, and definitive intervention within structured systems of care. The generation of prospective, shock-specific evidence—despite inherent challenges—will be essential to refine patient selection, optimize timing, and promote greater consistency in contemporary practice.
